# Supplementary material for: Are carbon emissions trading and green financial instruments synergistic? -Comprehensive quantitative research based on content analysis
Source: PLoS One. 2024 Mar 7;19(3):e0298601. doi: 10.1371/journal.pone.0298601 (PMC10919601; doi:10.1371/journal.pone.0298601)
Supplement: S1 Data — (ZIP) [file pone.0298601.s001.zip › Data/地级市Z_F工作报告.zip!/╔╜╢1⁄2/╫═▓⌐╩╨/└┤╫╘╣1⁄2╓┌║┼í╛┬φ┐╦╩2╛▌═°í┐_2013.docx]

各位代表：

　　现在，我代表市人民政府，向大会作政府工作报告，请予审议，并请各位政协委员和其他列席会议的同志提出意见。

　　一、2012年工作回顾

　　刚刚过去的一年，面对严峻的国际经济形势和繁重的改革发展稳定任务，全市上下在中共淄博市委的坚强领导下，牢牢把握主题主线和稳中求进的工作总基调，积极作为、攻坚克难，着力稳增长、调结构、促改革、惠民生，较好地完成了全年经济社会发展任务，殷实和谐经济文化强市建设迈出新步伐。

　　(一)努力克服经济下行压力，稳增长保发展取得新成效。本轮经济下行持续时间之长、影响范围之广，是多年来少有的。我们坚持把稳增长放在经济工作的突出位置，着力强化投资拉动，加强分类指导，加大要素保障，积极防控风险，千方百计保持全市经济在良性区间运行。预计全年地区生产总值将迈上3600亿元台阶，比上年增长10.5%左右；实现境内财政总收入518亿元，其中地方财政收入236.28亿元，增长16.06%；固定资产投资、社会消费品零售总额和外贸进出口总额分别增长20%、16%和6%；规模以上工业主营业务收入、利税、利润分别增长12%、6.5%和5%；金融机构各项存贷款余额分别突破3000亿元和2000亿元。

　　(二)着力加快转方式调结构，转型发展迈出新步伐。预计全年完成技改投资700亿元，增长28%；六大战略性新兴产业主营业务收入、利税、利润增幅分别高出规模以上工业9个、19个和18个百分点，高新技术产业产值占比达到28.1%。新增省级以上企业研发机构16家，其中国家级3家。品牌带动和标准化战略实现新突破。中心城区东部化工区搬迁改造基本完成，南部区域产业升级稳步推进。服务业投资占全部投资的比重达到49.2%，培育服务特色优势初见成效。现代农业发展步伐加快，粮食生产实现“十连增”，“三品一标”农产品达到257个，基层农技推广与服务体系、农产品质量安全监管体系和以水利为重点的支撑保障体系进一步健全。

　　(三)扎实推进新型城镇化，城乡统筹发展取得新进步。中心城区大外环以内260平方公里区域实现控规全覆盖。以市文化中心、华润城市综合体为代表的一批地标性工程，以晋豫鲁铁路沂源段、南水北调地方配套工程、坚强智能电网、西五路跨线桥为代表的一批基础设施工程，以清水润城、森林围城、生态园林城市为代表的一批生态建设工程顺利推进，城市承载辐射功能进一步增强。集中扶持14个省、市级示范镇建设发展，全面完成“两区三村”建设改造任务，改造农村公路317公里，新解决18万农村居民的饮水安全问题。环境保护工作力度不断加大，节能减排目标全面完成，国家森林城市创建工作正式启动，全市空气质量良好率比上年提高10个百分点。涌现出全国环保典型孟祥民，全社会生态文明理念进一步强化，生态文明建设不断加强。

　　(四)切实惠民生保稳定，社会建设取得新成绩。城乡居民人均收入分别增长13%和15%左右。财政总支出的56.83%用于民生建设，民生政策得到全面落实。新增城乡就业25万人，社会保障工作进一步加强。超额完成省下达的保障性住房建设任务。素质教育扎实推进，教育教学质量明显提高。医药卫生体制改革不断深化，公共卫生和基本医疗服务水平日益提高。人口自然增长率控制在2.9‰以内，低生育水平保持稳定。成功承办体操世界杯淄博站比赛，“十艺节”筹备工作有序推进。全国文明城市创建成果进一步巩固，双拥共建和优抚安置工作成效显著。深入实施固本强基维稳工程，圆满完成十八大安保等重大任务，切实加强安全生产和食品药品监管工作，完善应急管理体系，保持了社会大局和谐稳定，我市被评为“全国法治城市创建活动先进单位”。统计、物价、广播电视、民族宗教、外事、侨务、对台、防震减灾、气象、人防、史志、档案、妇女儿童、老龄、慈善、残疾人、红十字以及援藏、援疆等各项事业都实现了新发展，取得了新成绩。

　　各位代表，在过去的一年里，我们按照市委关于“发展发展再发展、实干实干再实干”的工作要求，在抓好常规工作的同时，着力在事关全局和长远的大事上动脑筋、求突破，集中精力抓了以下工作：

　　一是实施创新驱动发展战略，塑造新的产业竞争优势。深入实施新一轮传统产业技改计划和战略性新兴产业倍增工程，积极构筑以先进制造业和现代服务业为主导的现代产业体系。一批传统产业脱胎换骨，一批新兴产业呼之欲出，特别是以氯碱离子膜、高效无毒脱硝催化剂、非对称大动力电容电池等为代表的一批重大高新技术成果加速产业化，以文昌湖旅游度假区、华润城市综合体、太保电话运营中心等为代表的一批现代服务业项目加快推进，预示着淄博经济结构的战略性调整正在实现质的提升和突破。

　　二是努力扩大内需，增强经济增长的内生动力。面对外需萎缩的不利形势，我们注重强化内需拉动，着力扩投资、促消费。特别是去年四季度以来，进一步加大对上争取力度，切实强化资金保障，积极拓展用地空间，集中推出了今年上半年前具备开工条件、总投资656亿元的100项重点项目，与各大金融机构达成重点项目签约授信总额2277亿元。这批重点项目，投资规模大、技术含量高、预期效益好，对拉动经济增长、促进产业转型、提高经济竞争力、提升城市形象与功能，必将产生重大　推动作用。

　　三是充分放大自身优势，提高淄博的城市地位。着眼在全省区域发展大局中争取更大主动，我们全力加强淄博特有的重大开放创新平台建设。大力支持齐鲁股权托管交易中心规范发展、快速扩容，中心挂牌企业达到135家，直接和间接融资55亿元，全省性股权交易市场的影响力显著扩大，得到国家和省充分肯定。加快发展壮大现代物流业，积极支持淄博保税物流中心、正本物流保税仓库等拓展空间、提升功能，进一步增强了服务自身发展、辐射服务全省的能力。

　　四是大力推进改革开放，激发经济社会发展活力。出台了目标明确、导向鲜明的推动区县域科学发展的意见，确立倍增、振兴和跨越“三个板块”，大大激发了各级争先进位的积极性。积极创新“网格化”社会管理新模式，基层基础工作呈现新面貌。不断深化企业改革，全市有6家企业通过首发、借壳或增发，实现融资再融资54亿元。设立总规模20亿元、初始规模10亿元的淄博市产业发展基金，发行15亿元城运债。成功举办第十二届陶博会和第十一届新材料技术论坛。对外开放克服困难，在招商引智、提升现代制造业水平等方面呈现新的亮点，实际利用外资增长11.4%。其他各项改革稳步推进，地区发展活力显著增强。

　　在推动经济社会平稳健康发展的同时，我们始终高度重视加强政府自身建设。自觉接受人大及其常委会的监督和政协的民主监督，虚心听取各民主党派、工商联和社会各界的意见，认真办理人大代表建议和政协委员提案。深入贯彻实施《山东省行政程序规定》，扎实推进行政审批“两集中、两到位”改革，不断提高依法行政水平。严格落实党风廉政建设责任制，切实加强作风效能建设，树立了为民、务实、清廉的良好形象。

　　各位代表，过去一年全市经济社会发展取得的成绩来之不易。成绩的取得，得益于党中央、国务院和省委、省政府的科学决策与亲切关怀，得益于市委的坚强领导，得益于市人大、市政协与社会各界的有力监督与大力支持，得益于全市人民团结奋斗、拼搏实干。在此，我代表市人民政府，向全市广大人民群众，向各民主党派、工商联、各人民团体和社会各界人士，向驻淄部队、武警官兵、政法干警和中央、省驻淄单位，向海内外所有关心支持淄博发展的同志们、朋友们，表示衷心的感谢和崇高的敬意！

　　在充分肯定成绩的同时，我们也清醒地看到，我市经济社会发展还面临一些困难和问题，主要是：宏观环境不稳定不确定因素较多，经济下行压力依然较大；转方式、调结构任重道远，可持续发展面临的资源、环境和空间约束加大，产业竞争力和科技创新能力亟待提高；保障和改善民生任务繁重，统筹城乡和区域协调发展、加强和创新社会管理面临许多新课题。同时，经济社会发展对推进政府管理创新提出了新的更高要求。对这些困难和问题，我们高度重视，将采取更加扎实有效的措施，认真加以解决。

　　二、2013年目标任务和工作重点

　　今年是全面贯彻落实党的十八大精神的开局之年，是实施“十二五”规划承前启后的关键一年，是为全面建成小康社会奠定坚实基础的重要一年，做好今年工作意义十分重大。今年政府工作总的指导思想是：全面贯彻落实党的十八大精神，坚持以邓小平理论、“三个代表”重要思想、科学发展观为指导，紧紧围绕主题主线，坚持“强化生态文明、加快内涵发展”的总体思路和“发展发展再发展、实干实干再实干”的工作要求，聚精会神谋科学发展，凝心聚力促转调创新，着力保持经济持续健康发展，着力构建现代产业体系，着力促进“四化”同步发展，着力加强生态文明建设，着力推动文化大发展大繁荣，着力改善民生促进和谐，为建成更高水平、更高质量的全面小康社会奠定坚实基础。

　　今年全市经济社会发展的主要预期目标建议为：地区生产总值增长10.5%左右，公共财政预算收入增长12%左右，固定资产投资增长18%左右，社会消费品零售总额增长15%左右，实际利用外资和进出口总额均增长10%左右，城市居民人均可支配收入和农民人均纯收入均增长11%左右，人口自然增长率控制在3.3‰以内，城镇登记失业率控制在4%以内。上述指标安排，既考虑了与市第十一次党代会和“十二五”规划确定的目标相衔接，又综合考虑了今年经济社会发展的宏观环境、支撑条件以及发展趋势等要素，还兼顾了增加财政收入、扩大城乡就业、保障和改善民生等方面的要求，经过努力是完全可以实现的。

　　重点抓好以下八个方面的工作：

　　(一)在加快转型中实现经济持续健康发展。保持经济持续健康发展是一项长期性、全局性的重要战略任务。要按照党的十八大要求，加快转变经济发展方式，切实把推动发展的着力点转到提高质量和效益上来，实现尊重经济规律、有质量、有效益、可持续的发展。

　　大力强化转型发展的共识。历史积累形成的结构性矛盾突出、资源环境约束特别严重，是我们的特殊市情。当前，面对国内外环境倒逼我们扩大内需、提高创新能力、促进经济发展方式转变的新机遇，彻底改变和摆脱固有思维方式的束缚与路径依赖，实现新的转型发展，比以往任何时候都更为紧要和迫切。新的转型发展，就是符合党的十八大提出的“一个立足点”、“四个着力”、“五个更多依靠”要求的发展。要在全社会凝聚形成早转早主动、晚转则被动的广泛共识，不管环境如何变化、不管经济增长的压力大小，都始终紧紧扭住转变经济发展方式这条主线不放松，一抓到底，抓出成效。

　　在推进转型中实现持续健康发展。牢牢把握扩大内需这一战略基点，充分发挥投资的关键作用和消费的基础作用，着力提高经济增长的质量和效益。一是以优质投资增量引导转型发展。总的原则是，保持投速，扩大投量，优化投向，提高投效。既要牢牢坚持以项目建设为纲，把项目建设作为推动科学发展的总抓手，持续加大投资力度，保持经济增长强劲动力；又要切实优化投资结构，引导新增投资集中投向关键领域和薄弱环节。2013年，安排全市重大项目150项左右，主要涵盖现代农业、技术改造、高新技术、现代服务业、基础设施以及社会民生等重点领域，努力以投资结构的优化带动经济结构的优化，进而带动投资效益的提高，带动产业科技含量、附加值和竞争力的提高。绝对不能因为眼前利益、局部利益而再加重“两高一资”、产能过剩的包袱，加剧既有的重大结构性矛盾！二是抓住用好扩大消费的机遇。深化收入分配制度改革，释放居民消费潜力。把扩大消费与结构调整更好地结合起来，积极培育高端消费类品牌，扩大绿色节能环保产业优势，做大做强骨干商贸流通企业，改造提升大型专业批发市场，大力发展电子商务、连锁经营和物流配送，增强我市经济增长的内生动力。三是把经济发展的成果更加充分地体现到财税增加、居民增收上来。强化效益优先的财源建设导向，建立以税收为核心的企业绩效动态评价体系，以“纳税500强”企业为主体，培植壮大优质骨干财源。

　　建立健全有利于推动转型发展的体制机制。进一步加强产业政策、财政政策与金融政策的协同，加强结构调整规划、产业布局规划、城乡建设规划和生态建设规划的有机衔接和统一控制，确保全市转方式调结构沿着正确方向扎实推进。坚持区别对待、有保有压，凡是有可能“劣化”、加剧结构性矛盾的，坚决控住；凡是有利于显著优化产业结构、提升经济素质与效益的，全力保障。制定出台《加快重大项目建设的意见》，建立“要素资源跟着项目走”的保障机制，保证土地、信贷、财政支持等关键要素向全市重大项目集成配置；建立包括领导分级挂包、全程督促检查、定期考核通报制度在内的重大项目建设推进机制，确保重大项目顺利推进实施，尽快发挥效益。

　　(二)深入推进经济结构战略性调整。推进经济结构的战略性调整，事关经济增长的质量和效益，事关生态文明建设，事关淄博的核心竞争力。要加快构建现代产业体系，大力推进传统产业升级改造和战略性新兴产业培育，下大力气推动服务业快速跨越发展，积极发展现代农业，努力塑造淄博经济新的竞争优势。

　　加快工业结构优化升级。着力解决制约淄博工业由大变强的关键结构性问题，加快由工业大市向工业强市的跨越。今年，安排全市重点工业项目103项，总投资621亿元，其中技改投资和新兴产业投资占80%以上。一是以拉长产业链为重点，促进传统产业向高端快速提升。大力发展全产业链经济，把新一轮技改计划实施的重点，更多地转向围绕拉长石化、机电、陶瓷等主导产业链策划和实施项目，下大气力抢占产业链和价值链的高端，在基本不增加或较少增加资源消耗和排放的情况下，大幅度提高产业的附加值和竞争力。今后，原则上不再新上单纯扩大产能的传统产业项目。支持一批“两化融合”示范企业、示范项目，促进工业化与信息化深度融合。二是加速高新技术产业化，推动战略性新兴产业倍增发展。全力支持我市具备显著比较优势的关键技术研发和产业化项目，加速扩大氯碱离子膜、高效无毒脱硝催化剂、非对称大动力电容电池等一批革命性成果的产业化应用规模，着力走好园区化、集群化发展的路子，把新材料、精细化工、汽车及机电装备、新能源与节能环保装备、电子信息等产业发展成为千亿级产业集群，抢占制高点、形成竞争力。三是提升重点园区建设发展水平，推动产业集聚集约发展。对全市资源环境和生产力布局有重大影响的项目，必须坚持全市一盘棋，统筹规划，科学布局。要强化产业发展规划和建设用地规划的刚性约束，研究建立利益分配机制，促进相关行业企业打破行政区划界限，向最适宜发展的专业园区转移聚集。充分发挥高新区对全市高新技术产业发展的辐射带动功能，加快把齐鲁化工区打造成全市化工产业集约发展的重大平台，把东岳氟硅材料产业园建设成具有国际影响力的功能膜材料产业基地，努力形成一批千亿级特色产业园区。进一步明确各区县省级经济开发区产业定位，加快退城进园、退地进园、退村进园步伐，不断提高园区经济占区县域经济的比重。四是培育发展一批大企业、大集团。促进重要生产要素向核心竞争力强、成长性高、对地方发展贡献大的“纳税500强”、“创新发展500强”等骨干企业集中配置，支持其上新创新、上市挂牌、兼并重组、做大做强。重视发挥中央、省驻淄大企业优势，促进企地融合发展。全面落实支持小微企业发展的财税、金融政策，加快中小企业服务体系建设，增强区域经济活力。

　　加快现代服务业提质增速。淄博工业化、城镇化水平较高，现代服务业发展的潜力和空间巨大。要坚持扩大规模和提升层次并重，加快实施现代服务业跨越发展工程，努力使服务业增速明显高于工业，切实改变服务业占比过低的被动局面。今年安排全市服务业重点项目110项，总投资995亿元，确保服务业增加值占比提高1.5个百分点以上，力争提高2个百分点。一是优先发展生产性服务业。深刻把握现代产业融合发展的新趋势，大力发展与石油化工、新材料等主导产业关联度高、特色鲜明、竞争优势强的现代物流业，培育一批重点物流园区和大型物流企业，推动淄博保税物流中心向综合保税区过渡，把淄博建设成为名副其实的鲁中物流“旱码头”；大力发展对提高产品附加值和竞争力有关键作用的工业设计，加强与国内外高水平设计机构合作，积极引进高端设计人才和团队，支持相关院校培养专业设计人才，办好“创意淄博”工业设计大赛，全面提高工业设计水平。加快发展科技信息、技术咨询等科技服务业以及律师仲裁、会计税务等为企业经营和社会管理服务的中介服务业，加快形成新的增长点。二是做大做强金融服务业。以规划建设金融商务区、齐鲁股权大厦为带动，加快建设淄博金融聚集区。举全市之力加快齐鲁股权托管交易中心发展，力争到“十二五”末，实现中心挂牌企业500家以上，总市值1000亿元以上，融资总额500亿元以上，进一步提升全省性股权交易市场的影响力。健全支持金融发展的政策体系，优化金融生态环境，深化政银企合作。认真贯彻执行稳健的货币政策，努力创新金融产品，扩大信贷规模，降低融资成本，满足实体经济需求。三是下大气力提升壮大文化旅游业。淄博文化旅游资源丰厚，要创新思路，提升境界，高水平、大手笔地规划建设一批具有重大品牌影响力和市场效应的高端项目，显著提高淄博旅游的吸引力和综合效益。全面加快文昌湖旅游度假区开发建设步伐，切实搞好齐文化和聊斋文化旅游的整体系统策划与开发，抓好周村古商城5A级景区创建工作，推进淄博旅游集散中心建设，完善城市旅游功能，把文化与生态资源优势转化为旅游产业发展优势。四是促进房地产及相关服务业健康发展。认真落实国家房地产调控政策，有效保护刚性住房需求，合理引导住房消费。积极发展商业地产，搞好华润城市综合体等一批重点项目建设，发展新型业态，促进消费升级。针对老龄化快速发展的实际，制定专门规划和优惠政策，加快推进养老社区建设，完善社会养老服务体系。全面落实各级支持服务业发展的政策措施，加强规划引导，放宽市场准入，构建多元化投融资体制，加快培育一批服务业领军企业和知名品牌。

　　扎实推进农业现代化。坚持工业反哺农业、城市支持农村和多予少取放活方针，在工业化、城镇化互动发展中同步推进农业现代化，促进农业持续增效，农民持续增收。一是大力发展高效特色农业。深入实施小麦、玉米高产创建工程，力争粮食生产再获丰收。加快推进蔬菜、畜牧、果业、林木花卉等高效特色农业标准化基地建设，提升都市农业示范园发展水平，培育名优农业品牌，提高农业综合效益。搞好农产品质量安全监督检测体系建设，加强绿色生产，从源头上保证农产品质量安全。二是不断提升农业物质技术装备水平。大兴农田水利，抓好国家中小河流治理规划项目、小型病险水库除险加固和引黄灌区续建配套工程建设，确保年内南水北调地方配套工程具备通水条件。加快推进农村饮水安全工程，全面完成山丘区找水打井任务，再解决15万农村人口的饮水问题。实行最严格的水资源管理制度，确保供水安全、防洪安全、生态安全。着力强化科技兴农，完善基层农技推广服务体系，加强农村实用人才培训工作，加快良种工程和农业机械化、信息化建设，提高设施农业、精准农业发展水平，提高科技对农业的贡献率。三是加快推进农业经营机制创新。深化农村改革，积极探索新型农业生产经营模式，大力发展农民专业合作和股份合作，发展多种形式的适度规模经营。实施农民专业合作社提升工程和骨干农业龙头企业集群工程，构建集约化、专业化、组织化、社会化相结合的新型农业经营体系。发挥供销社连接城乡的桥梁纽带作用，畅通农产品进城和工业品下乡双向流通渠道。四是全面落实强农惠农富农政策。广辟就业门路，拓宽增收渠道，确保农民收入与城镇居民收入同步增长，并力争增长更快一些。坚持和完善农村基本经营制度，依法维护农民承包经营权、宅基地使用权、集体收益分配权，壮大集体经济实力。确保各项支农补贴及时足额到位。深入实施新十年扶贫开发规划，启动十万贫困农户脱贫奔康工程，抓好首批60个重点贫困村财政扶贫资金建设项目，让广大农民平等参与现代化进程、共同分享现代化成果。

　　(三)大力实施创新驱动发展战略。把创新驱动发展作为面向未来的重大战略，全面落实鼓励创新的一系列政策措施，加快科技创新、产品创新、品牌创新、产业组织创新、商业模式创新，全面提升我市核心竞争力。

　　加快构建富有活力的技术创新体系。深化科技体制改革，紧紧围绕科技与经济紧密结合这个核心，加快建立企业为主体、市场为导向、产学研相结合的技术创新体系。深入实施企业创新能力提升工程，突出企业在技术决策、研发投入、产学研合作和成果转化中的主体地位，着力培育提升一批国家级、省级企业研发中心、重点实验室、院士工作站、博士后科研工作站和产业技术创新联盟，切实增强企业创新能力。实现创新发展必须依靠创新型企业，创新型企业关键要靠创新型企业家。要举办“创新大讲堂”，开拓领导干部和企业家视野，提高整体创新意识和上新创新的能力。

　　积极推动科技成果转化平台和商业模式创新。一方面，要构建富有效率的创新转化平台，从我市最具比较优势的新材料等产业入手，依托高新区现有基础，加快建设国内一流、国际有一定影响力的开放式创新中试基地，配套搞好相关公共服务设施，吸引海内外相对成熟的领先科技成果来淄博中试和就地产业化；另一方面，要构建富有活力的投融资机制，市级产业发展基金重点支持创新型企业和高新技术产业化项目，充分借助齐鲁股权托管交易中心这一特有平台，畅通创新市场与资本市场有机对接的渠道，大力引进和培育风险投资，为中试基地和全市创新型企业的快速成长提供最适宜的资本土壤，努力使“一基地”、“一中心”成为带动全市高新技术产业发展的“发动机”、“加速器”。

努力营造鼓励创新创造的良好环境。搞好国家知识产权示范市建设，深入实施标准化战略，提高知识产权创造、应用和保护水平。坚持人才优先、以用为本，加大创新要素参与分配的力度，大力培养和引进高层次创新创业人才，引导人才向科研生产一线流动聚集。设立发明创新奖励基金，对年度重大专利和发明持有人进行奖励，引导形成全社会关注创新、支持创新、参与创新的浓厚氛围。

　　(四)积极稳妥推进新型城镇化。城镇化是现代化进程中的一个大战略。未来五到十年，将是城镇化大发展、大跨越的重大机遇期。要抢抓机遇、发挥优势，努力开创我市新型城镇化发展新局面。

坚持以“人的城镇化”为核心，塑造新型城镇化新格局。目前，我市城镇化率已达64%以上，下一步，要突出以“人的城镇化”为核心，切实把城镇化发展的重点放在提升质量和内涵上。进一步深化户籍管理制度改革、土地管理制度改革，着力提高户籍人口城镇化率。加快完善城乡一体化发展体制机制，着力在城乡规划、基础设施、公共服务等方面推进一体化，促进城乡要素平等交换和公共资源均衡配置，形成以工促农、以城带乡、工农互惠、城乡一体的新型工农关系、城乡关系。加快教育、医疗、住房保障等基本公共服务覆盖全部城镇常住人口，切实缩小城乡发展差距，实现农业转移人口市民化。

　　努力提质提速，提高城市现代化水平。淄博的城市建设正处于蓄势待发、孕育突破的关键时期，要坚持高起点规划、高标准建设、高效能管理，加快打造“新淄博、大都市”。一是以淄博新区建设为龙头推进“四个层级”协调发展。推动新城区重要功能区块建设全面提速，在继续抓好文化中心等在建工程的同时，抓紧启动公共资源交易中心、社会管理指挥调度中心以及商务区、金融区和休闲公园等重点工程建设，加快把淄博新区打造成为新的城市发展增长极和体现现代都市形象的核心区。切实加强对中心城区大外环以内260平方公里区域的规划控制，注重增强主城区与副中心城区在规划、建设、管理上的整体性、协调性和关联性，增强辐射带动小城镇及中心村发展的能力。二是加强重要基础设施建设。集中组织实施一批对提升城市功能有重大作用的交通、能源、环保重点工程，积极推进省道102淄博段改建、寿平铁路淄博段、联通路综合整治、火车站南广场建设以及淄博环保热电厂、城区供热管网“汽改水”、坚强智能电网等一批重点项目，提高承载辐射功能。三是切实提高城市运营管理水平。建立健全“全时段、全覆盖”的城市环卫保洁机制和城乡垃圾一体化处理体系，推进城市管理的数字化、网格化、精细化。将资本运作、资产运营的理念贯穿于城市规划、建设和发展的全过程，保障城市可持续发展。

　　坚持城乡一体化，大力加强小城镇建设。以14个省、市级示范镇为重点，整体推进、重点突破，着力提高小城镇产业支撑能力和基础设施承载能力，使之成为拉动经济发展的重要增长极和带动农村劳动力就近转移的重要平台，为城乡一体化发展创造条件。一是高水平搞好小城镇规划建设。按照社区园区同步规划、三次产业协调发展的要求，尽快高水平完成小城镇总体规划和控制性详细规划编制修订工作，引导产业集聚、土地集约和人口集中。按照统筹兼顾、适度超前的原则，加强小城镇基础设施建设，精心设计建设核心街区、重要建筑群，着力建设一批小城镇精品工程。二是提高小城镇产业支撑能力。着力在发展特色产业集群、建设产业园区和培植骨干企业上下功夫，省、市级示范镇地方财政收入年均增幅要分别达到25%和20%以上，实现有活力、有后劲、可持续的发展。坚持集约、智能、绿色、低碳的方针，支持示范镇建设特色园区，积极承接城市劳动密集型企业转移，同时为城市高端产业发展腾出空间。三是加大对小城镇建设发展的扶持力度。全面落实扩权强镇政策，科学修编示范镇土地利用规划，新增建设用地指标和增减挂钩指标优先向示范镇倾斜，拓展示范镇建设发展空间。在落实省扶持政策的基础上，由市、区县财政共同支持，给予每个省、市级示范镇每年1000万元和600万元的专项扶持。实行市大班子领导挂包和市直部门对口帮扶制度，建立完善对示范镇的考核评价体系，确保示范镇建设发展走在全省前列。

　　提高区县域科学发展水平。在“四个层级”城镇化体系中，区县的地位和作用至关重要。要认真贯彻落实省市关于加快区县域科学发展的决策部署，促进区县域科学发展，争先进位。一是明确“三个板块”发展目标。“十二五”期间，张店区、临淄区、桓台县和高新区作为倍增板块，经济总量和财政收入要实现倍增，为全市发展作出更大贡献；淄川、博山、周村三个老工业区作为振兴板块，要确保实现“十二五”规划确定的目标，力争发展得更快、更好一些；高青、沂源两个欠发达县作为跨越板块，经济总量和财政收入要实现翻番，努力形成后发优势。鼓励各区县开展与省内外先进区县对标活动，明确目标、提升标杆、奋力赶超。二是有效激发动力活力。认真落实扩大区县域经济管理权限、税收分成、超收奖励等政策，在融资、用地等方面加大支持力度。健全完善重点突出、导向鲜明的考核评价体系，强化对财税收入、有效投入和科技创新的考核，激发各区县竞相发展、争先进位的积极性。三是用足用好重大政策利好。以高水平策划实施项目为切入点，积极争取“蓝黄两区”扶持政策以及产业投资基金项目。主动对接省会城市群经济圈发展规划，最大限度用好资源枯竭型城市、沂蒙革命老区等政策，继续做好博山独立工矿区政策争取工作，务求政策效益最大化。

　　(五)大力加强生态文明建设。党的十八大首次把生态文明建设列入中国特色社会主义建设总体布局，这是在发展理念和实践上的重大创新。要把生态文明建设融入经济、政治、文化、社会建设的各方面和全过程，努力建设美丽淄博、生态淄博。

　　扎实推进节能减排。深入实施“十二五”节能减排综合性工作方案，多还老账、不欠新账，努力实现绿色发展、循环发展、低碳发展。一是突出源头治理。扎实推进“四大节能改造工程”和主要污染物减排工程，对现有重点用能企业和排放大户进行限期治理改造。深入推进农业、交通、公共机构、商业与民用等领域节能降耗，大力发展绿色建筑。坚持不懈地淘汰落后产能，坚决关停能耗高、污染重以及不具备安全生产条件的“土小”企业。加强产业发展趋势预测，做好过剩产能化解工作。以全省钢铁行业重组为契机，推动我市钢铁产业科学整合、提升转型。二是严格增量管控。严格落实能耗和排污总量控制以及能评、环评等制度，严把新建项目节能环保准入门槛，严格执行环保“第一审批权”和“一票否决权”，坚决杜绝新上高耗能、高排放、高污染和高危项目。三是推动集约发展。大力推动相关产业向专业园区聚集，解决布局散乱和污染围城、污染扩散问题。加快齐鲁化工区等重点园区的循环经济改造，深入实施中心城区南部区域产业优化升级工程，促进产业集约发展和资源高效利用。

　　加大环境保护工作力度。以创建国家环保模范城为抓手，集中抓好水和大气环境污染防治。继续实施骨干河道综合整治，加快新扩改建一批城镇污水处理厂、人工湿地和中水回用工程，完善雨污分流管网，搞好100家重点企业的水污染深度治理，主要河流水质稳定达标。加强重点企业脱硫脱硝设施建设，大力推进清洁能源替代工程，巩固提升秸秆禁烧转化成果，研究制定PM2.5排放削减办法，全市空气质量良好率达到65%以上。大力加强农村面源污染治理和重金属污染防治，加快建设全市危险废物综合处置中心，健全环境应急管理体系，确保环境安全。进一步完善公安环保一体化执法机制，对环境违法行为坚决予以“顶格处罚”。

　　大力加强城乡生态环境建设。突出抓好国家森林城市、国家生态园林城市创建工作，深入实施森林围城等大环境绿化工程，进一步增加城乡绿量、提升绿化品质，全年完成造林合格面积10万亩以上，新增城市绿地300公顷以上。加强森林防火能力、有害生物防控能力建设，确保森林资源安全。扎实开展全国水生态文明城市创建工作，搞好重点流域生态环境综合整治，推进“清水润城”工程，构建生态水系。加快建立生态补偿机制，加强饮用水水源地保护。加大太河水库、文昌湖、马踏湖、大芦湖、四宝山等重点区域的生态修复力度，深化露天开采矿山环境综合治理。坚持最严格的耕地和基本农田保护制度，扎实开展增减挂钩、工矿废弃地复垦利用、低丘缓坡等未利用地开发利用试点，积极争创全国国土资源节约集约模范市。进一步完善推进生态淄博建设的法规体系、标准体系和目标责任考核体系，建立生态文明建设长效机制。

　　(六)扎实推进文化强市建设。文化是城市综合实力和竞争力的重要组成部分。要适应人民群众日益增长的精神文化需求，全面促进文化大发展大繁荣，加快由文化大市向文化强市的跨越。

加强社会主义核心价值体系建设。坚持社会主义先进文化的前进方向，深入开展社会主义核心价值体系宣传教育普及，用社会主义核心价值体系引领社会思潮、凝聚社会共识。大力弘扬民族精神和时代精神，进一步培育以诚信、务实、开放、创新为主要特征的城市精神。深入开展道德领域突出问题专项教育和治理，深化群众性精神文明创建活动，巩固提升全国文明城市创建成果。

　　加强公共文化服务体系建设。按照公益性、基本性、均等性、便利性的原则，加快构建覆盖城乡、惠及全民的基本公共文化服务体系。加快镇(办)综合文化站、农村文化大院、镇村公共电子阅览室和社区文化中心建设，提高文化惠民工程实效。积极做好“十艺节”承办、参演工作，搞好有关场馆设施改造，打造一批艺术精品。扎实推进文化遗产保护，加强齐文化研究开发，进一步扩大齐文化品牌影响力。

　　提升文化产业竞争力。坚持以改革创新为动力，大力推进文化与经济社会融合发展。积极培育发展一批文化龙头企业，实施一批具有示范效应和产业拉动作用的文化产业重点项目，加快发展动漫游戏、印刷出版、工艺美术和文博、创意会展等特色产业。深入推进国有文艺院团改革，组建淄博演艺集团，增强经营活力。加强文化市场和网络社会管理，净化市场环境。

　　(七)坚定不移深化改革扩大开放。坚持把改革创新精神贯穿到经济社会发展各个环节，不失时机地推进重点领域改革，实施更加积极主动的开放战略，切实增强转型发展的动力与活力。

深化重点领域改革。处理好政府与市场的关系，更加尊重市场规律，更好地发挥政府作用。坚持两个“毫不动摇”，激发各类市场主体的活力和创造力。进一步放宽市场准入，鼓励和扩大民间投资，大力发展和引进股权投资基金。完善各类国有资产管理体制，积极推进国有资源整合重组，加大优质资产注入力度，增强市城运公司投融资功能，提高政府通过市场化手段优化资源配置的能力。改进支持产业发展的模式，科学整合建设性财政资金，集中用于对重点项目进行贴息，充分放大财政资金的导向和杠杆效应。进一步深化企业改革，完善支持企业上市的政策措施，力争新增上市公司3家。加快地方金融改革创新，鼓励小额贷款公司增资扩股，规范发展信用担保业，不断完善金融服务体系。建立健全监测预警、组织协调和应急处置机制，坚决守住不发生系统性和区域性金融风险的底线。

　　提高开放型经济发展水平。面对世界经济进入深度转型调整期的新形势，要因势利导，顺势而为，促进开放型经济向着优化结构、拓展深度、提高效益的方向转变。一是坚持招商引资规模与质量并重。瞄准我市新型工业化、城镇化的重点需求，集中力量实现产业链招商、产权招商、新城区重点功能区块招商和企业境外上市的新突破。加大招才引智力度，鼓励支持有条件的企业积极引进高端人才和专业人才，走快捷可靠的创新发展之路。继续办好陶博会和新材料技术论坛，将其打造成为我市开放创新的重要平台。二是坚持对外经贸总量与结构并举。全面落实国家鼓励出口的政策，深入实施品牌战略和科技兴贸战略，推动加工贸易向产业链高端延伸，不断提高机电和高新技术产品出口比重。充分发挥淄博保税物流中心的口岸服务功能，加快建设综合保税物流园区。努力扩大重要产品出口，扩大先进设备、关键零部件和能源原材料大宗商品进口，适度扩大消费品进口。三是进一步做好“走出去”的文章。支持宏达矿业等境外投资和资源开发项目，鼓励企业建立境外自主营销渠道，切实用好两个市场、两种资源，拓展经济发展空间。积极参与区域经济合作，加快石嘴山淄博工业园等“飞地”园区建设。

　　(八)加强民生和社会建设。按照“守住底线、突出重点、完善制度、引导舆论”的思路，扎实做好民生工作，加强和创新社会管理，多谋民生之利，多解民生之忧，促进社会和谐稳定。

　　统筹做好城乡就业和社会保障工作。实施就业优先战略和更加积极的就业政策，重点做好以高校毕业生为重点的青年就业工作和农村转移劳动力、城镇困难人员、退役军人就业工作，促进以创业带动就业。力争年内新增城镇就业8.3万人，新增农村劳动力转移就业7万人。推行企业工资集体协商制度，保障职工劳动权益。着力完善社会保障体系，抓好各项保险的扩面征缴工作。进一步提高城镇职工医疗保险统筹基金最高支付限额、提高城镇职工和居民大额医疗救助基金最高支付限额、提高医保门诊统筹报销比例，将城镇居民医疗保险和新农合财政补助标准提高到每人每年280元，将农村低保标准提高到年人均不低于2500元，积极稳妥开展城乡居民大病保险。加强农村“五保”供养和城镇“三无”人员、孤残儿童保障以及流浪乞讨人员救助工作。大力发展慈善、红十字事业，健全残疾人社会保障和服务体系。

　　加强保障性住房建设。保质保量完成省政府下达我市的保障性住房建设任务，年内开工建设各类保障　性住房8000套，年底前基本建成10000套。结合产业布局调整以及全市资本市场、创新平台和重点园区发展需要，坚持政府主导、企业主体、市场运作，着重推进公共租赁住房建设，稳定职工队伍，吸引留住人才。用好住房公积金贷款支持保障性住房试点政策，推动在普通商品房项目中配建保障性住房。认真贯彻廉租房、经适房、公租房三个《管理办法》，建立健全住房保障准入、退出和管理机制，确保分配公平、管理规范、群众满意。

　　提高社会事业发展水平。以办好人民满意的教育为目标，全面完成学前教育三年行动计划、中小学校舍安全工程和办学条件标准化建设任务，科学推进中心城区东部城区教育资源布局调整，加快淄博三中新校等学校建设，构建现代职业教育体系，支持发展高等教育，完善教育管理机制，促进各级各类教育协调、均衡发展。深化医药卫生体制改革，巩固完善基本药物覆盖制度和基层运行新机制，推进临淄区、桓台县公立医院综合改革试点。启动市全科医师临床培养基地等一批重点医疗卫生项目建设，不断增强公共卫生和医疗服务能力。加强计划生育工作，稳定低生育水平，提高出生人口素质。广泛开展全民健身运动，组织办好第16届市运会。加强国防动员和双拥共建工作，推动军民融合式发展。保障妇女、儿童、老年人合法权益，促进老龄、社会福利等各项事业健康发展。

　　加强和创新社会管理。全面实施社会管理创新“三大工程”，加快构建“六位一体”立体化、全覆盖社会管理新格局，夯实基层基础工作。深入实施第四个社区建设三年规划，大力推行网格化管理和村(居)民事务代办制度。科学整合管理资源，加快建设市社会管理调度指挥中心，积极争取国家“智慧城市”试点，构建全域数字城市，实现对社会治安、安全生产、城市管理、环境保护、规划建设等重点领域的全覆盖、网格化、即时化管理，从本质上提高社会管理的信息化、现代化水平。坚持以群众工作统揽信访工作，认真排查调处影响发展稳定的突出矛盾和问题。深入实施固本强基维稳工程，积极开展普法依法治理，推进公共安全体系建设，严厉打击违法犯罪活动。大力加强安全生产工作，加强食品药品安全监管，健全应急管理体制机制，努力维护社会大局和谐稳定。

　　三、建设干事创业的服务型政府

　　经济社会发展的新形势和人民群众的新期待，对政府工作提出了新的更高要求。必须坚持为民、务实、清廉，切实加强干事创业的服务型政府建设，大力唱响发展发展再发展、实干实干再实干的主旋律。

　　强化宗旨意识，建设服务政府。人民对美好生活的向往，就是我们的奋斗目标。我们要牢记全心全意为人民服务的宗旨，真正把以人为本、执政为民作为检验政府一切工作的最高标准。坚持深入基层，深入群众，问政于民、问计于民、问需于民，凡是有利于富民强市的事情，全力去做；凡是国家出台的惠民政策，坚决落实；凡是群众反映强烈的突出问题，务必解决。切实以对淄博发展的真责任，对城乡百姓的真感情，密切与人民群众的血肉联系，交出一份合格的答卷。

　　大兴实干之风，建设效能政府。空谈误国，实干兴邦。要大力精简会议文件，下决心改进会风文风，杜绝庸懒散奢，聚精会神出实招、干实事、求实效。实行“两条线”工作法，常规工作明确分工、高效推进，重点工作全力以赴、务求突破。集中力量抓好促发展创优势的大事，抓好打基础管长远的要事，抓好破瓶颈增后劲的难事，千方百计增强淄博综合实力，提升淄博城市地位。强化工作目标责任制，严格督查考核，严明工作纪律，确保令行禁止、政令畅通，全面提高政府执行力。

　　推进依法行政，建设法治政府。认真落实依法治国基本方略，提高各级政府运用法治思维和法治方式深化改革、推动发展、化解矛盾、维护稳定的能力，全面提高行政决策、执行、监督的法治化、民主化、科学化水平。加强行政程序能力建设，深化行政审批“两集中、两到位”改革，提高电子政务水平，规范行政执法行为，改革行政复议体制，推动政府管理与服务创新，优化发展环境。自觉接受人大及其常委会监督、政协民主监督、媒体舆论监督和社会监督，确保权力在阳光下运行。

　　坚持从严治政，建设廉洁政府。全面落实党风廉政建设责任制，健全完善惩治和预防腐败体系，加强对重要岗位、关键环节的监督和制约，强化领导干部经济责任审计，规范权力运行。推进反腐倡廉制度创新，加快建设公共资源交易平台，全面推行廉政风险防范管理。各级政府及政府各部门领导干部要带头执行《廉政准则》，严格执行重大事项报告制度，以勤政为民、廉洁清正的实际行动，赢得群众的信任与支持，维护党和政府的良好形象。

　　各位代表！面对全市人民的信任和重托，面对新的挑战和考验，我们倍感使命在肩、责任重大。让我们紧密团结在以习近平同志为总书记的党中央周围，在中共淄博市委的坚强领导下，解放思想、励精图治，万众一心、埋头实干，奋力开创淄博科学发展新局面，为加快建成更高水平、更高质量的全面小康社会而努力奋斗！
